# Supplementary material for: Fibromyalgia and Depression in Women: An 1H-NMR Metabolomic Study
Source: Metabolites. 2021 Jun 30;11(7):429. doi: 10.3390/metabo11070429 (PMC8304744; doi:10.3390/metabo11070429)
Supplement: Supplementary file 1 [file metabolites-11-00429-s001.zip › metabolites-1227908-Supplementary.pdf]

metabolites

MDPI

Supplemetary Materials

Fibromyalgia and Depression in Women: an 1H-NMR Metabolomic Study

Carmen Marino [1,†], Manuela Grimaldi [1,†], Paola Sabatini [2], Patrizia Amato [3], Arianna Pallavicino [1] and Carmen Ricciardelli [1] Anna Maria D'Ursi [1,*]

[1] Department of Pharmacy, University of Salerno, Via Giovanni Paolo II, 84084 Fisciano, Italy; cmarino@unisa.it (C.M.); magrimaldi@unisa.it (M.G.), carmen.ricciardelli@gmail.com (C.R), ariannapallavicinogmail.com (A.R)
[2] U.O.C. Clinical Pathology D.E.A. III Umberto I, Nocera Inferiore, Italy; paola.sabatini1@gmail.com
[3] Ser.T, Cava dei Tirreni ASLItaly; patriziaamato277@gmail.com
* Correspondence: dursi@unisa.it
† These authors contributed equally to this work.

Citation: Marino, C.; Grimaldi, M.; Sabatini, P.; Amato, P.; Pallavicino, A.; Ricciardelli, C.; D'Ursi, A.M. Fibromyalgia and Depression in Women: An 1H-NMR Metabolomic Study. *Metabolites* **2021**, *11*, 429. https://doi.org/10.3390/metabo11070429

Academic Editor: Ilaria Campesi and Margherita Ruoppolo

Received: 3 May 2021
Accepted: 23 June 2021
Published: 30 June 2021

Publisher's Note: MDPI stays neutral with regard to jurisdictional claims in published maps and institutional affiliations.

Copyright: © 2021 by the authors. Licensee MDPI, Basel, Switzerland. This article is an open access article distributed under the terms and conditions of the Creative Commons Attribution (CC BY) license (http://creativecommons.org/licenses/by/4.0/).

Table of contents:

Table S1. Important features identified by Fold Change and logarithmic Fold Change (log2(FC)) parameters calculated.
Table S2. Important features identified by t-tests values, p-values (Threshold <0.05), logarithmic p-values and False Discovery Rate (FDR) parameters calculated for the most statistically significative compounds.
Table S3. PLS-DA classification of the five different components (comps) based on accuracy, R2, Q2 related to MVA analysis between fibromyalgic patient and controls derived by NMR analysis.
Table S4. PLS-DA classification of the five different components (comps) based on accuracy, R2, Q2 related to MVA analysis between healthy controls, patients with moderate depression and patients with severe depression
Figure S1. ROC curve of biomarker. The sensitivity is on the y-axis, and the specificity is on the x-axis. The area-under-the-curve (AUC) is in blue. On the right: Box-plot of the concentrations of the selected feature between controls (0) and fibromyalgic patients (1) within the dataset. A horizontal line is in red indicating the optimal cutoff.
Figure S2. PLS-DA score plot (a) and loading plot (b) for ^{1}H NMR data collected in CPMG spectra using 19 controls (0, red), 13 fibromyalgic with moderate depression (1, green) and 6 fibromyalgic with severe depression (2, blue).
Figure S3. ROC curve of biomarker. The sensitivity is on the y-axis, and the specificity is on the x-axis. The area-under-the-curve (AUC) is in blue. On the right: Box-plot of the concentrations of the selected feature between fibromyalgic patients with moderate depression (1) and fibromyalgic patients with severe depression (2) within the dataset. A horizontal line is in red indicating the optimal cutoff.

Table S1. Important features identified by Fold Change and logarithmic Fold Change (log2(FC)) parameters calculated.

Compounds	Fold Change	log2(FC)
D-Glucose	9491	13.212
Hypoxanthine	0.018267	-5.7746
Acetic acid	0.043038	-4.5382
2-Hydroxybutyrate	0.05329	-4.23
Formic acid	0.078331	-3.6743
Creatinine	0.083113	-3.5888
Betaine	0.11026	-3.181
Pyruvic acid	0.12985	-2.9451
L-Arginine	0.15953	-2.6481
Citric acid	0.17055	-2.5518
Acetone	0.1726	-2.5345
Lysine	0.17357	-2.5264
L-Carnitine	0.19325	-2.3715
Succinic acid	0.21386	-2.2252
L-Tryptophan	0.22884	-2.1276
Creatine	0.22977	-2.1217
L-Threonine	4.2693	2.094
L-Proline	0.25091	-1.9948
Choline	0.25796	-1.9548
L-Tyrosine	0.29669	-1.753
L-Glutamine	3.3603	1.7486
1-Methylhistidine	0.30426	-1.7166
L-Alanine	0.30953	-1.6919
L-Methionine	3.1338	1.6479
3-Hydroxybutyric acid	0.33956	-1.5582
L-Lactic acid	2.8553	1.5137
L-Serine	0.35904	-1.4778
Glycine	2.6022	1.3797
L-Leucine	0.39029	-1.3574
Isopropanol	0.40604	-1.3003
Isobutyric acid	0.41498	-1.2689
Dimethylsulfone	0.41507	-1.2686
L-Isoleucine	0.4163	-1.2643
L-Phenylalanine	0.42085	-1.2486
Acetoacetate	0.44488	-1.1685
Ethanol	0.46156	-1.1154
L-Asparagine	0.47853	-1.0633

Table S2. Important features identified by t-tests values, p-values (Threshold <0.05), logarithmic p-values and False Discovery Rate (FDR) parameters calculated for the most statistically significative compounds.

Compounds	t.stat	*p* value	-log10(p)	FDR
D-Glucose	23.886	2.4297e-32	31.614	1.0691e-30
2-Hydroxybutyrate	-19.618	8.6876e-28	27.061	1.9113e-26
Citric acid	-17.021	1.2113e-24	23.917	1.7765e-23
L-Tryptophan	-14.386	4.1261e-21	20.384	4.5387e-20
L-Carnitine	-11.442	1.0219e-16	15.991	8.9929e-16
1-Methylhistidine	-11.266	1.9364e-16	15.713	1.42e-15
Creatinine	-10.755	1.2631e-15	14.899	7.9393e-15
Hypoxanthine	-10.693	1.5935e-15	14.798	8.7645e-15
L-Proline	-10.495	3.3239e-15	14.478	1.625e-14
L-Isoleucine	-9.9348	2.7364e-14	13.563	1.204e-13
Creatine	-9.7799	4.9305e-14	13.307	1.9722e-13
L-Leucine	-9.2732	3.4344e-13	12.464	1.2593e-12
Formic acid	-8.499	6.9344e-12	11.159	2.347e-11
Betaine	-8.3485	1.2492e-11	10.903	3.9259e-11
Pyruvic acid	-8.3233	1.3787e-11	10.861	4.0442e-11
Acetic acid	-8.2342	1.955e-11	10.709	5.3764e-11
L-Threonine	8.1348	2.8861e-11	10.54	7.47e-11
L-Phenylalanine	-8.0818	3.5545e-11	10.449	8.6889e-11
L-Methionine	7.8233	9.8121e-11	10.008	2.2723e-10
Choline	-7.7509	1.3046e-10	9.8845	2.8702e-10
Succinic acid	-7.4395	4.4429e-10	9.3523	9.3089e-10
L-Arginine	-7.0983	1.7004e-09	8.7694	3.4009e-09
Lysine	-5.9093	1.7409e-07	6.7592	3.3304e-07
L-Tyrosine	-5.8268	2.3874e-07	6.6221	4.3768e-07
Acetone	-5.6939	3.9621e-07	6.4021	6.9733e-07
3-Hydroxybutyric acid	-5.5916	5.8399e-07	6.2336	9.883e-07
Isobutyric acid	-5.531	7.3428e-07	6.1341	1.1966e-06
L-Alanine	-4.5076	3.0967e-05	4.5091	4.8663e-05
L-Glutamine	4.4109	4.343e-05	4.3622	6.5894e-05
Methanol	-3.7052	0.0004623	3.3351	0.00066364
L-Valine	-3.7017	0.00046756	3.3302	0.00066364
Acetoacetate	-3.4081	0.0011739	2.9304	0.0016142
L-Asparagine	-3.3644	0.0013414	2.8724	0.0017886
Isopropanol	-3.2656	0.0018076	2.7429	0.0023393
L-Serine	-3.2086	0.0021422	2.6691	0.0026931
L-Glutamic acid	-2.9805	0.0041511	2.3818	0.0050736
Glycine	2.8752	0.0055804	2.2533	0.0066362
L-Aspartic acid	-2.6556	0.010125	1.9946	0.011724

Table S3. PLS-DA classification of the five different components (comps) based on accuracy, R2, Q2 related to MVA analysis between fibromyalgic patient and controls derived by NMR analysis.

	**Comp1**	**Comp2**	**Comp3**	**Comp4**	**Comp5**
Accuracy	1.0	1.0	1.0	1.0	1.0
R2	0.94	0.98	0.98	0.99	0.98
Q2	0.93	0.95	0.95	0.93	0.92

Table S4. PLS-DA classification of the five different components (comps) based on accuracy, R2, Q2 related to MVA analysis between healthy controls, patients with moderate depression and patients with severe depression.

	**Comp1**	**Comp2**	**Comp3**	**Comp4**	**Comp5**
Accuracy	0.84	0.76	0.71	0.73	0.74
R2	0.79	0.85	0.88	0.90	0.93
Q2	0.75	0.66	0.65	0.62	0.59

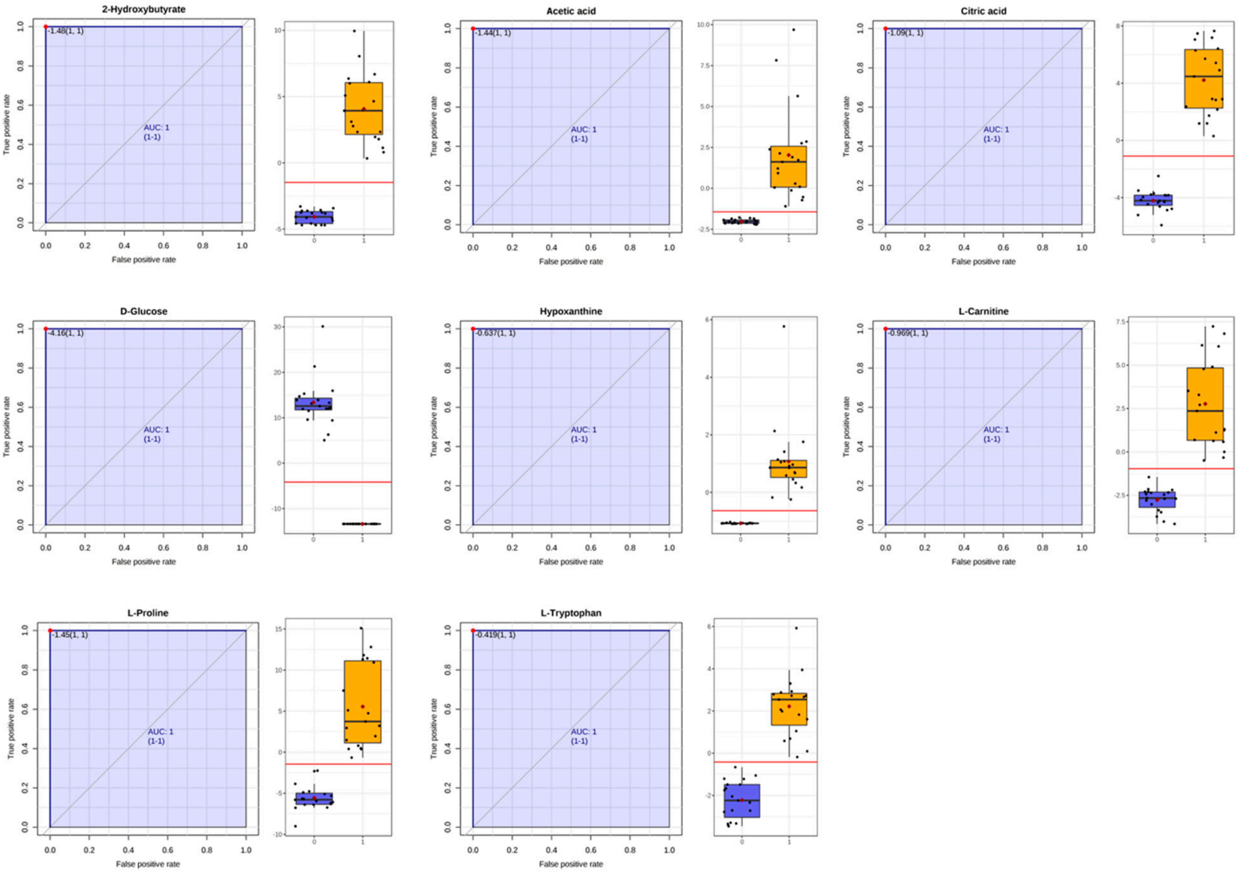

Figure S1. ROC curve of biomarker. The sensitivity is on the y-axis, and the specificity is on the x-axis. The area-under-the-curve (AUC) is in blue. On the right: Box-plot of the concentrations of the selected feature between controls (0) and fibromyalgic patients (1) within the dataset. A horizontal line is in red indicating the optimal cutoff.

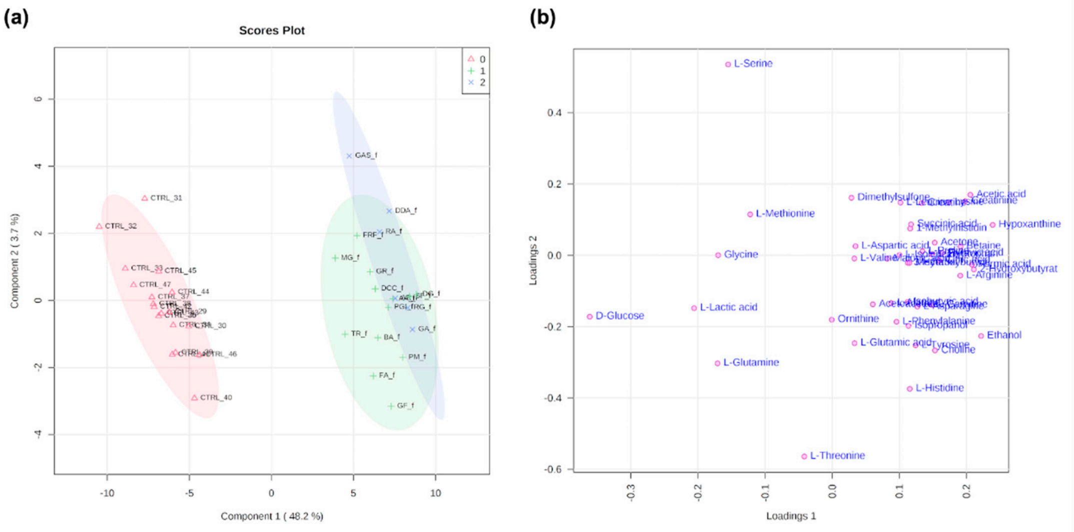

Figure S2. PLS-DA score plot (a) and loading plot (b) for ^{1}H NMR data collected in CPMG spectra using 19 controls (0, red), 13 fibromyalgic with moderate depression (1, green) and 6 fibromyalgic with severe depression (2, blue).

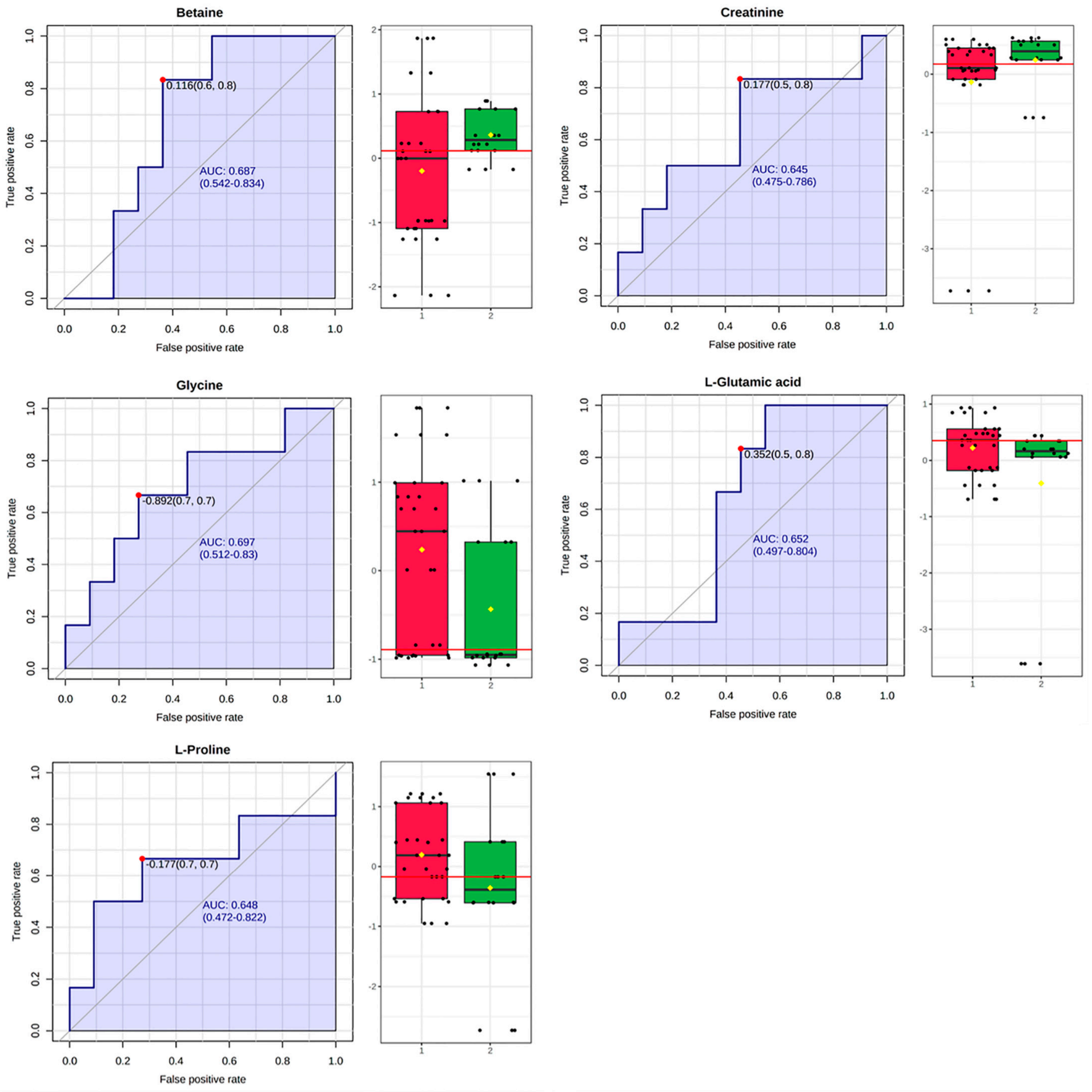

Figure S3. ROC curve of biomarker. The sensitivity is on the y-axis, and the specificity is on the x-axis. The area-under-the-curve (AUC) is in blue. On the right: Box-plot of the concentrations of the selected feature between fibromyalgic patients with moderate depression (1) and fibromyalgic patients with severe depression (2) within the dataset. A horizontal line is in red indicating the optimal cutoff.
